# The Use of Telehealth Technology in Assessing the Accuracy of Self-Reported Weight and the Impact of a Daily Immediate-Feedback Intervention among Obese Employees

**DOI:** 10.1155/2011/909248

**Published:** 2011-06-15

**Authors:** Nicolaas P. Pronk, A. Lauren Crain, Jeffrey J. VanWormer, Brian C. Martinson, Jackie L. Boucher, Daniel L. Cosentino

**Affiliations:** ^1^HealthPartners, Minneapolis, MN 55440-1309, USA; ^2^JourneyWell, Minneapolis, MN 55425, USA; ^3^HealthPartners Research Foundation, Minneapolis, MN 55440-1524, USA; ^4^Harvard School of Public Health, Boston, MA 02115, USA; ^5^Minneapolis Heart Institute Foundation, Minneapolis, MN 55407, USA; ^6^Cardiocom Multi-Disease Management, LLC, Chanhassen, MN 55317, USA

## Abstract

*Objective.* To determine the accuracy of self-reported body weight prior to and following a weight loss intervention including daily self-weighing among obese employees. *Methods.* As part of a 6-month randomized controlled trial including a no-treatment control group, an intervention group received a series of coaching calls, daily self-weighing, and interactive telemonitoring. The primary outcome variable was the absolute discrepancy between self-reported and measured body weight at baseline and at 6 months. We used general linear mixed model regression to estimate changes and differences between study groups over time. *Results.* At baseline, study participants underreported their weight by an average of 2.06 (se = 0.33) lbs. The intervention group self-reported a smaller absolute body weight discrepancy at followup than the control group. *Conclusions.* The discrepancy between self-reported and measured body weight appears to be relatively small, may be improved through daily self-monitoring using immediate-feedback telehealth technology, and negligibly impacts change in body weight.

## 1. Introduction

Assessment of self-reported body weight is widely regarded as being more practical than obtaining a measured weight [1]. In both community samples and employed populations, self-reported weight has been shown to be fairly accurate [2–5]. On the other hand, among certain populations such as younger women [6, 7], a greater tendency to underestimate body weight has been observed. Likewise, substantial underestimates have been reported among people who are obese or are otherwise attempting to lose weight [8–10] or restrain their eating [8, 11]. Generally speaking, the heavier the individual, the greater the self-reported bias toward an underestimate of body weight [8, 10]. In the workplace setting, this bias phenomenon can be especially problematic because self-reported body weight may call into question the utility of employee self-assessments in estimations of program impact. In addition, recruitment and screening efforts are complicated when seemingly eligible participants are misclassified based on inaccurate self-reported weight [12].

Relatively little is known about what can improve the accuracy of self-reported body weight in obese populations. Experimental evidence suggests that a (clinically) measured body weight taken prior to completing a self-report questionnaire improves the accuracy of self-reported body weight [9, 13]. A cross-sectional survey by Flood and colleagues [14] found that women who self-weighed at least weekly were more likely to report their body weight correctly as compared to those who self-weighed less frequently. Taken together, this evidence seems to suggest that if a person “weighs-in” before self-reporting their body weight, especially if weigh-ins occur frequently, the more accurate their self-reported body weight will be. Unfortunately, none of these studies focused specifically on obese populations, where the concern over self-reported body weight accuracy seems especially relevant in the context of interventions. Furthermore, recent advances in telehealth technologies can be used to obtain unbiased measured body weight using electronic scales that, using visual and audio options, can provide immediate feedback. Hence, the purpose of this study was to test the effect of frequent self-weighing, a key behavioral weight loss strategy [15, 16], on the accuracy of self-reported body weight among employed, obese adults participating in a randomized-controlled trial.

## 2. Methods

### 2.1. Participants

Participants were enrollees in the Weigh-By-Day study, a randomized controlled weight management trial among obese employees. Although the main results of this study have been described elsewhere [17], the research presented in the current paper specifically refers to the use of telehealth technology in assessing the accuracy of self-reported body weight prior to and following weight loss. The number of participants recruited into the study was based on a power analysis for the main study on weight loss to detect differences between conditions at an *α*-level of 0.05. Inclusion criteria for study participants were the following: age, 18 years or older, obese (body mass index (BMI) ≥ 32 kg/m^2^), actively employed, and willingness to perform daily self-weighing. Exclusion criteria for the study included concurrent participation in other weight-loss programs, currently receiving cancer treatment, pregnancy, or inability to speak/read English. The HealthPartners Research Foundation Institutional Review Board approved the study protocol, and all participants signed a written informed consent to participate.

### 2.2. Conditions

Participants completed baseline questionnaires and attended an orientation session where measured weights and heights were obtained and random assignment to the two study conditions was performed using a computer-controlled block randomization list designed to allocate 45% and 55% of the study sample to the immediate-start (intervention group) and delayed-start group (control group), respectively. Hence, both groups received a weight loss intervention although the control received the intervention 6 months after the enrollment in the study. As a result, the first 6 months of the study provide an opportunity to test the impact of the telehealth technology (described below) on the accuracy of self-reported weight prior to and following a period of active weight loss including daily self-weighing.

Participants in the intervention group received a telephone-based behavioral weight loss intervention similar to that described by Jeffery and colleagues [18]. The intervention included a written treatment manual, behavior change tools such as a food/activity log and pedometer, and up to 10 phone-based health coaching calls from a registered dietitian and/or health educator.

Participants in the intervention group also received a home telehealth scale (Thin-Link, Chanhassen, Minn, USA) to use for 6 months. The intervention group participants were instructed to weigh themselves daily, and the telehealth scale provided visual and audio feedback on weight following each weigh-in. Each day, as participants would step on the scale, the device also prompted them to answer a series of basic questions on weight loss behaviors (e.g., “Did you exercise today?”). The device was connected to an analog telephone line via an internal modem that would immediately transmit information on each participant's measured weight and responses to the behavioral questions directly to the health coaching team. In addition, if a participant did not show any weigh-in data or gained more than 4 lbs within a 3-day period, an alert was generated that prompted the health coach to consider a proactive outreach and connect with the participant based on customized, objective data. Before actual health coaching calls, the health coaches would review the participant's progress and provide them with personalized feedback and suggestions. 

Participants in the delayed-start group represent the control condition in this analysis. The control group did not receive the home telehealth scale or the telephone-based behavioral weight loss program during the six-month period of this study.

### 2.3. Measures

In-person assessments were conducted at baseline and 6, 12, and 18 months after-baseline. Since the control group initiated treatment after the first six months of the study (delayed-start), only the baseline and 6-month follow-up timepoints are used in this analysis. The body weights used in the analysis of this research (both measured and self-report) at baseline and 6 months refer to those obtained during the in-person assessments only, that is, they do not reflect the daily weighing that was part of the intervention group's use of the telehealth device. During the in-person assessments at baseline and 6 months, measured body weight was obtained using calibrated Thin-Link scales for all participants in both the intervention and the control groups. Self-reported body weights were obtained from screening questionnaires that were completed prior to the assessment of measured body weight during the in-person visit. 

The main outcome of interest was the discrepancy between self-reported and measured body weight. Two forms of weight discrepancy, absolute (the primary outcome) and relative (the secondary outcome), were assessed. Absolute weight discrepancy was defined as the absolute value of the difference between self-reported and measured weight. It is a useful analytical measure because it provides a precise estimate of the magnitude of the weight discrepancy (i.e., how far are the self-reported and measured weights away from each other?). Relative weight discrepancy, on the other hand, is calculated by subtracting self-reported body weight from measured body weight. It is a useful descriptive measure because it provides a conceptually simple estimate of the direction of the weight discrepancy (i.e., is self-reported body weight higher or lower than measured body weight?).

### 2.4. Analysis

*t*-tests and *χ* ^2^ tests were used to compare differences in baseline characteristics. General linear mixed model regression models were estimated using SAS PROC MIXED (time within participant, random participant intercepts, unspecified covariance structure, and restricted maximum likelihood estimation) to test whether relative or absolute weight discrepancy was different across study groups, between baseline and followup, or differentially over time by study group. Absolute and relative weight discrepancies were predicted separately from measurement time (baseline, followup), which varied within participants, and randomized treatment group (Intervention, Control), which varied across participants. The mixed model approach was chosen because, relative to a general linear model approach (e.g., repeated measures ANOVA or ANCOVA), it readily accommodates variation in the number of repeated observations per participant and accurately estimates standard errors of parameters without reliance on imputation to replace missing observations. All analytical procedures were computed using SAS 9.1 (SAS Institute, Cary, NC, USA), and an *α*-level of 0.05 was used as the criterion for statistical significance.

## 3. Results

Following recruitment and screening for eligibility in the study, 138 individuals were invited to the study orientation. Of those invited, 38 did not attend, cancelled, or were confirmed ineligible, and, as a result, 100 participants were randomly assigned to the intervention (*n* = 45) or control group (*n* = 55). At 6-month followup, 87%  (*n* = 39) and 84%  (*n* = 46) of participants in the intervention and control groups, respectively, completed the in-person assessments.  Table 1 presents descriptive characteristics of the study population by study groups. Randomization was successful in that there were no significant differences between groups on any of the baseline measures as reported elsewhere [17]. 

Table 2 presents the absolute and relative mean weight discrepancies by study group at baseline and at followup. The top portion of Table 2 displays the means and standard errors that were calculated using all available data points at baseline and followup. The lower portion of Table 2 displays the model predicted means and standard errors from the separate mixed models in which weight discrepancy (either absolute or relative) was predicted from study group and measurement time. The absolute weight discrepancy variable indicates that participants tended to self-report a weight value that was on average 3.46 (se = 0.25) pounds different from what was measured (average discrepancy calculated across all subjects at baseline and followup). The relative weight discrepancy variable shows that participants tended to self-report a weight value that was on average 2.06 (se = 0.33) pounds less than what was measured (average discrepancy calculated across all subjects at baseline and followup). There were no baseline differences between the intervention and control groups with respect to absolute weight discrepancy, *P* = .35. The absolute discrepancy between measured and self-reported weight did not change over time (*P* = .32), nor did the rate of change over time differ across the study groups, *P* = .31. However, the simple effect of intervention versus control was significantly different at the 6-month follow-up measurement (*P* = .036).

A similar pattern of results emerged for relative weight discrepancy. The relative weight discrepancy was similar across study groups at baseline (*P* = .49) and did not change over time (*P* = .84), and the rate of change was similar across study groups (*P* = .41). For the relative weight discrepancy data, none of the simple effect comparisons were statistically significant. 

## 4. Discussion

The results of this study indicate that, in relative terms, the discrepancy between self-reported and measured body weights among obese employees is small, consistent over time, and not influenced by a self-monitoring intervention, thereby supporting the contention that self-reported body weight may be considered a reasonable estimate of measured body weight and body weight change among obese employees. In addition, participants who received a regular self-weighing intervention with immediate feedback and daily monitoring were significantly more accurate in their self-reported weight at followup. 

Cash and colleagues [13] observed considerable improvement in the accuracy of self-reported weight following just a single weigh-in. Flood et al. [14] observed more accurate self-reports among participants who practiced self-weighing just weekly. In the Cash et al. study, however, measured body weights were taken immediately before self-reported body weight was recorded. This may suggest that the delay between measured body weights relative to subsequent self-report of body weight heavily influences recall bias. In other words, it may be that the closer a measured body weight is taken relative to self-reported weight, the more accurate that self-reported body weight will be. This phenomenon may operate largely independent of self-weighing frequency, although this notion was not studied explicitly.

Consistent with previous research, participants in our study reported that they weighed less than what was observed on the scale. The magnitude of underreporting in our study was about 30 percent less than that observed in some weight loss programs [9, 19] and slightly higher compared to other more recent data [5]. As in the Dekkers et al. study [5], participants in this study self-reported their body weight relatively accurately from the start, thereby leaving less room for improvement. It may also be that participating in a research trial versus a workplace program alters the expectation that weights will be formally measured in the presence of others (i.e., researchers), which tempers the body image-related self-presentation bias that is suspected to underlie weight underreporting [13, 20].

Convenience and low cost make the use self-reported body weight an attractive solution for ongoing program administration. Self-reported weight may be questionable because it may result in underclassification of people into overweight and obese categories [5, 12]. In a weight loss intervention, however, where the primary outcome of interest is usually a relative variable (i.e., weight change), it seems to be more acceptable, at least in the short-term or during the active treatment phase [18, 19]. The relative discrepancy across intervention group participants in our study moved from about 3 lbs underreported at baseline to about 2.7 lbs underreported at followup. This relatively small change of about 0.3 lbs is due in part to the relatively small baseline discrepancy between self-reported and measured body weight. Furthermore, the comparison between baseline and followup involves self-reported body weights at both measurement points. As such, a consistent error may be biased toward a lower body weight cross-sectionally but allows for a relatively stable change estimate. Consider the following application of the data generated from the current analysis of the Weigh-By-Day trial data: participants in an employer-sponsored weight loss program have an average measured body weight of 200 lbs and an average self-reported body weight of 197 lbs at baseline. After six months of treatment (with or without regular self-weighing), the average measured body weight is 190 lbs and the average self-reported body weight is 188 lbs. Thus, the average measured body weight loss was 10 lbs while the average self-reported body weight loss was 9 lbs. Although the precise amount of weight loss would be slightly biased, it seems to be a reasonable estimate if resource constraints make it difficult to obtain measured weight.

A unique aspect of this study involved the novel use of a telehealth device that allowed for a direct observation of self-weighing behavior as well as the direct measurement of body weight itself. The participants were fully aware that the health coaches had knowledge of their behavior (self-weighing) as well as their body weight itself. Measured and self-reported body weights were completed prior to and following the weight loss intervention. These study components allowed us to measure the accuracy of self-reported body weight in a cross-sectional manner as well as following a self-weighing program intervention. 

The results of this study indicate that the accuracy of self-reported body weight, among obese employees appears to be relatively close to measured body weight, may be improved through daily self-monitoring, and negligibly impacts change in body weight. Despite the fact that directly measured weights will always be considered more accurate, the results of this study indicate that self-reported weights among obese employees may be considered acceptable for program effectiveness monitoring in real world applications which will greatly enhance program efficiency and affordability. Strategies to promote self-weighing may support improvements in accuracy of self-reported body weight and such efforts may be especially relevant since self-weighing has shown to be an evidence-supported recommendation for successful body weight loss, weight regain prevention, and prevention of weight gain among adults [16, 21].

## Figures and Tables

**Table 1:** Descriptive characteristics of all participants at baseline.

	Intervention	Control	*P*
*n*	45	55	
Age (years)	44.5 (1.4)	47.7 (1.1)	.065
Female	93.3%	89.1%	.461
Hispanic ethnicity	2.2%	0%	.266

Race			
American/Alaskan native	0.0%	5.5%	
Black/African American	8.9%	5.5%	
Native Hawaiian/Pacific Islander	2.2%	0.0%	.373
White	84.4%	87.3%	
Multiracial	2.2%	1.8%	
Unknown or unspecified	2.2%	0.0%	
Non-Hispanic White	84.4%	87.3%	.685

Marital status			
Never married	15.6%	12.7%	
Married, living with partner	71.1%	67.3%	.576
Divorced	13.3%	16.4%	
Widowed	0.0%	3.6%	

Education level			
High school diploma or GED	4.4%	3.6%	
Technical or associate's	28.9%	25.5%	
Some college	26.7%	18.2%	.688
Bachelor's degree	26.7%	40.0%	
Graduate degree	13.3%	12.7%	

Annual household income			
15,000–29,999	0.0%	1.9%	
30,000–44,999	20.0%	13.0%	
45,000–59,999	13.3%	13.0%	.873
60,000–74,999	13.3%	16.7%	
75,000–89,999	20.0%	22.2%	
90,000+	33.3%	33.3%	

Measured weight (lbs)	238.7 (5.7)	227.0 (4.4)	.102
Body mass index (kg/m^2^)	39.2 (0.9)	37.6 (0.6)	.152
Self-reported weight (lbs)	237.3 (5.7)	224.7 (4.3)	.078
Ideal weight (lbs)	154.3 (3.1)	154.0 (2.7)	.943
Ever dieted to lose weight (yes)	100%	94.6%	.112
Weight loss program participation in last 2 years (yes)	37.8%	56.4%	.064

Values are reported as mean ± standard error or frequency (% of column total).

**Table 2:** Change in absolute and relative weight discrepancy by study group over time.

	Intervention		Control	
	Baseline	Followup	Baseline	Followup
Observed				
Absolute weight discrepancy (lbs)	3.01 (0.37)	2.69 (0.37)	3.66 (0.44)	4.37 (0.76)
Relative weight discrepancy (lbs)	1.41 (0.55)	2.59 (0.39)	2.02 (0.61)	2.28 (0.94)

Model predicted				
Absolute weight discrepancy (lbs)	3.01 (0.52)	2.69 (0.56)	3.67 (0.47)	4.31 (0.52)*
Relative weight discrepancy (lbs)	1.41 (0.67)	2.54 (0.72)	2.05 (0.61)	2.21 (0.67)

Values are reported as mean ± standard error.

*Intervention versus control is *P* < .05 at followup.
